# The Undesirable Aliens Bill

**Published:** 1905-04-29

**Authors:** 


### The Hospital.

A Journal of

The Medical Sciences and Hospital Administration.

Vol. XXXVIII.—No. 970. SATURDAY, APRIL 29, 1905.

### The Undesirable Aliens Bill.

The expressed determination of the Prime Minister that the Undesirable Aliens Bill, which was read a first time in the House of Commons immediately before the recess, shall on this occasion be pushed forward with the whole strength of the Government, and practically as a measure by which they will stand or fall, has been received with very general satisfaction by the country. The Bill of last session, conceived upon much the same lines, was talked out by the Opposition on grounds which seemed to display a remarkable want of perception alike of the necessities of the public, of the currents of prevailing opinion, and of the generally familiar fact that circumstances alter cases. It is no doubt historically true that England has been greatly indebted, in former times, to the immigrants who have been driven to her shores by persecution; to the Flemings for the cloth manufacture of the West, to the French Huguenots for the silk weavers of Spitalfields, and so on through many examples; and it is also true that the asylum which she has from time to time afforded to the friends of political liberty has served to keep alive its sacred fire under many discouragements and through many dangers. But the immigrants to whom such statements would apply were persons of a totally different character from those whom the present Aliens Bill would exclude, and there is no real analogy between the cases. The Flemings and the Huguenots were not only skilled workmen, but peaceful and law-abiding citizens of the countries from which they were expelled, and would have enriched those countries by their industry, just as they ultimately enriched that which gave them shelter. The friends of political liberty had not yet learnt that its principles were to be promoted by the bomb of the anarchist or the dagger of the assassin; and would have turned away in utter disgust from many of those who now masquerade under the sacred name. The influx from Southern Russia and from other continental countries, against which our government is tardily devising measures of defence, consists largely of persons who would be a nuisance and a danger to any community among which they were distributed. It is of no avail for us to endeavour to elevate our working classes, to provide them with better habitations, to train them into habits of cleanliness, and to give them facilities for education and for amusement, if we suffer them to be overwhelmed by an influx of foreigners to whom vermin hunting is a pastime, and to whom what would be dire poverty in the estimation of a decent Englishman represents a degree of affluence of which they had not previously dreamed. It is of no avail for us to enforce the principles of sanitation among our own people, if we suffer these principles to be violated by the immigrants in every way and in every direction. It is not too much to say that the "undesirable aliens" of the last few years alone have put back the clock of progress in East London by something like half a century. At least that time will be required before those who have already been admitted, and their descendants, become sufficiently educated, civilised, and cleansed, to be fit to occupy a position of equality among even the humblest of our own countrymen.

It cannot be said that the Bill introduced by the Government errs on the side of undue severity; for it shows traces, in more than one of its provisions, of some inclination to be startled at shadows. But the evils against which the Bill is directed are, in effect, the importation of dirt, vermin, poverty, inefficiency, and disease; and it will at least have the good effect, as soon as it becomes law, of causing it to be known in foreign countries that our doors are no longer absolutely open to all who choose to pass through them. In the first instance there will presumably be a rush, for it is not proposed that the Bill, if passed, should come into operation until January 1st, 1906, and there will therefore be a period of some months, probably five or six, during which we must be prepared for desperate efforts to augment the foreign population to which we have so much reason to object. Perhaps the best method of dealing with this intervening period would be by the rigorous enforcement of all existing laws against filth and overcrowding, and against the concealment or domestic treatment of contagious disease; so that, if we must be prepared to endure a final influx of vagabonds, as many of them as possible may be consigned to hospitals and gaols. In the former they will at least be restrained from inflicting farther injury upon their neighbours; and in both they will be brought into a state of at least transient cleanliness and freedom from entomological colonies. But for the self-styled "friends"

of the working-man, who on this, as on every other occasion, have shown themselves to be his worst enemies, an Undesirable Aliens Act would at the present moment be in operation, and the working classes of East London directly, and of all parts of the kingdom indirectly, would have been saved from the additions made during the last few months to the number of the immigrants who live in luxury where an Englishman would starve, who keep down wages, who supply the police and the magistracy with continual employment, who lower the respectability and standard of character of the working classes as a whole, and who spread disease and filth wherever the contamination of their presence extends.

